# Chitosan coated cotton cloth supported zero-valent nanoparticles: Simple but economically viable, efficient and easily retrievable catalysts

**DOI:** 10.1038/s41598-017-16815-2

**Published:** 2017-12-05

**Authors:** Fayaz Ali, Sher Bahadar Khan, Tahseen Kamal, Khalid A. Alamry, Abdullah M. Asiri, Tariq R. A. Sobahi

**Affiliations:** 1 Center of Excellence for Advanced Materials Research, King Abdulaziz University, P.O. Box 80203, Jeddah, 21589 Saudi Arabia; 2 Department of Chemistry, King Abdulaziz University, P.O. Box 80203, Jeddah, 21589 Saudi Arabia

## Abstract

A simple, economically viable and fast method has been utilized for the preparation of highly active metal nanoparticles (MNPs) in coating layer of chitosan (CH) over cellulose microfibers of cotton cloth (CC). 2 wt% of CH solution was used for the coating of CC strips (CC-CH), and were kept in aqueous solutions of metal salts to adsorb metal ions. The CC-CH templated with metal ions were then treated with aqueous solution of NaBH_4_ to reduce the metal ions into zero-valent metal nanoparticles (M^0^). The CC-CH strips loaded with M^0^ were characterized by XRD, XPS, ATR-FTIR, FE-SEM and TGA, which indicates the successful synthesis of MNPs by this method. The M^0^/CC-CH strips were used as an efficient catalyst for the model reduction reaction of nitrophenol and toxic organic dyes. Among all the prepaped samples, Fe/CC-CH showed good catalytic activity for 4-NP and Rh-B dye reduction in the presence of NaBH_4_ with rate constants of 0.2937 min^−1^ and 0.3804 min^−1^, respectively. Moreover Fe/CC-CH has good catalytic reduction ability for MO and MB having rate constants equal to 0.1698 and 0.2802 min^−1^, respectively. Beside the good catalytic ability, it could be easily recoverable as compared to other available techniques. The recovery was completed by simply pulling the strip from the reaction matrix after completion of the reaction and can be used several times.

## Introduction

In recent years, the development of new catalytic system for the conversion of toxic chemicals into fine chemicals has become a foremost research area. The rational design of active, durable and selective catalysts is essential which can be tested on hazardous chemicals conversion. The use of catalyst in liquid phase can aid substantially in the recovery, separation and reuse of catalyst and also required afford for product separation from reaction matrix, thus major contribution on the synthetic procedure is required for environmental performance.

Recently, metallic nanoparticles (MNPs) have attracted much attention from the researchers community because of their various applications in variety of fields, *i.e*, sensing, bio-sensing, antimicrobial coating, drug delivery and so-on. Beside these common applications, MNPs are extensively applied as catalysts in various chemical reactions such as Suzuki-cross-coupling^1^, hydrogen liberation^2^, hydrocarbon oxidation^3,4^, phenolic derivative upgradation reactions^5^ and ring opening^6^. MNPs have high activity due to their small sizes which originates from their high surface to volume ratio. Most of the MNPs can removed different pollutants due to their electron donating tendency including the anions through reduction. Due to these properties, a considerable attention of the environmental researchers has been attracted towards the metal nanoparticles^7–9^. Therefore, most of MNPs applied for removal of toxic chemicals including: Fe, Cu, Co, Ag, Ni, Ti, Pd, Pt and Al^8,10,11^. Two main hurdles are associated with the practice of MNPs as a catalyst. Firstly, agglomeration of nanoparticles readily occurs due to van der Waals forces, and prevention of these agglomerate is difficult. Such agglomeration of nanoparticles drops the catalytic activity because it results in decrease of surface area^12^. Secondly, the separation of nanoparticle is difficult after completion of reactions because of their small size^13^. Researcher focused their efforts to overcome the above-mentioned hurdles. They suggested that the single solution was to utilize catalytic supports to prevent agglomeration and easy recycling of nanoparticles. The usage of catalyst support has two-fold benefits. Firstly, they improve the dispersability and stability and secondly, the separation and reuse of catalyst from the reaction matrix become easy^14,15^. Nanoparticles can be assembled or supported on high surface area materials, and the resulting composite materials can be used as a catalyst for model reaction.

Cotton cloth (CC) is well known porous and flexible fabric made from natural cotton fibers, which has high porosity and hierarchical network structure with functional groups such as hydroxyl groups forming complicated surface morphology^16,17^. For instance, each cotton fiber is composed of multiple individual cotton fibrils, which are in turn composed of multiple micro-fibrils bundled together^16,18^. The micro-fibrils are made of poly-D-glucose chains, which are usually arranged in partially crystalline domains^16^. This structure allows the fiber to absorb large amounts of water and other polar solvents, which causes the cotton fibers to swell when placed in a solution.

Chitosan (CH) is a polysaccharide which is derived from the deacetylation of chitin. Chitin is abundantly available in nature and commonly found in many invertebrates and in the cell wall of most algae and fungi^19^. The physical, chemical and functional characteristics make it to be considered as an incredible and versatile polymer. The advantageous properties of CH include it’s; biodegradability, biocompatibility, cationic nature, good adsorption capacity, film-forming capabilities, adhesive characteristics, permeability-enhancing effect and many more, and is considered as cost-effective and safe^20,21^. However, it is often dissolved in acidic medium because it’s not completely soluble in water and thus limit its application in various fields. Despite of its solubility shortcomings, it has been applied in various fields and in many industries, such as; biotechnology, medical, pharmaceutics, cosmetic, food and nutrition, water engineering, ophthalmology, paper technology, photography and others^20,22,23^. The availability of free amino groups in its structure provide various modification possibilities which can be further functionalized to increase capacity for metal ions uptake^24–27^.

From the past few years, synthetic polymer-based catalyst supports were synthesized, and catalytic efficiencies of various metal nanoparticles were analyzed^25,28,29^. The hydrogels were exposed as good supports for catalyst in aquatic chemical reactions because in this case the catalyst surface is exposed to the reactant molecules^30,31^. However, hydrogels are soft and break during handling. Therefore, to avoid the long procedure for the designing and preparation of new materials, we relied on abundantly available natural resources such as cotton^17,18^ and CH (derivative of chitin)^29,32^ as catalytic support.

Herein, we present a facile and cost-effective method to synthesize MNPs on CC-CH as a catalyst for water treatment. The M^0^/CC-CH were made by CH aqueous solution coating on CC, adsorption of metal ions (M^+^) from their respective aqueous salt solutions followed by treating the samples with NaBH_4_ solution to convert M^+^ to their respective zero-valent metal nanoparticles (M^0^). The synthesized samples were characterized by using FT-IR, XRD, XPS, TGA and FE-SEM. The M^0^/CC-CH were applied as dip-catalysts in chemical reduction of nitrophenols and azo dyes. These azo dyes and nitrophenols are highly toxic and many struggles have been made which exploited the proficient catalysts to transform them into non-toxic substances. The nano-zero-valent iron (Fe) nanoparticles loaded on CC-CH exhibited high catalytic efficiency for the conversion of these toxic substances. This novel Fe/CC-CH has great potential to apply as an environmentally friendly, economical, easily recyclable and sustainable catalyst.

## Experimental

### Materials

Chitosan in the form of yellowish powder with a degree of deacetylation >75% and high molecular weight was purchased from Sigma-Aldrich, Ireland. Sodium borohydride (NaBH_4_, 99%), acetic acid, ferrous sulphate heptahydrate (FeSO_4_.7H_2_O, 98%), nickel sulphate heptahydrate (NiSO_4_.7H_2_O, 99%), copper sulphate pentahydrate (CuSO_4_.5H_2_O, 98.5%), cobalt chloride hexahydrate (CoCl_2_.6H_2_O), 4-nitrophenol (4-NP) and 2-nitrophenol (2-NP) were purchased from BDH chemicals, England. Silver nitrate (AgNO_3_) was purchased from MERCK. Rhodamine-B (Rh-B), methylene blue (MB) and methyl orange (MO) dyes were purchased from Koch-lite laboratories, England. All chemicals were of analytical grade and utilized without any further purification. This work was carried out with water having resistivity of 18.2 MΩ cm.

### Catalyst synthesis

The following procedure of two-steps was implemented for the synthesis of catalyst

1. The yellowish chitosan (CH) solution was prepared by dissolving 2% w/v of CH in dilute acetic acid aqueous solution (20%v/v) by stirring overnight at room temperature. It was then coated on cotton cloth (CC) by dipping the rectangular pieces (0.4 × 2 cm^2^) of CC in this solution for 10 min. The excess solution was wiped out from the pieces of CC and kept for drying.
2. MNPs were synthesized inside CH layer on CC by adsorption of metal ions followed by their reduction with NaBH_4_. As mentioned above that pieces of CC were treated with CH solution to form CC-CH strips. Then CC-CH strips were kept in separate aqueous solutions of metal salts of FeSO_4_.7H_2_O, NiSO_4_.7H_2_O, CuSO_4_.5H_2_O, CoCl_2_.6H_2_O and AgNO_3_ for 4 hours, which turned the white CC-CH strips to yellowish, greenish, irish blueish, reddish and brownish respectively. The strips were then gently washed with deionized water, and introduced into 100 mL of 0.1 M NaBH_4_ aqueous solution. The black color was observed as soon as these strips were introduced into NaBH_4_ aqueous solution. The strips were remaining dipped for 30 min in NaBH_4_ solution to completely reduce the metal ions into zero-valent metal nanoparticles (M^0^). The strips of CC-CH loaded with MNPs (M^0^/CC-CH) were washed with deionized water after this step, and freshly used in further analysis.

### Characterization

Crystal structure of the samples were analyzed by using X-ray diffraction (XRD) on PANalytical diffractometer with a Cu Kα radiations (λ = 0.154 nm) source. The XRD was operated at 40 KV and 50 mA, and data were recorded at a scan rate of 2° 2θ min^−1^. The crystallinity percentage (Cr%) were calculated from the XRD patterns of pure CC, CC-CH, and MNPs loaded on CC-CH samples, using the following equation (1),

1 $${\rm{Cr}} \% =\frac{{I}_{(200)}-{I}_{amorphous}}{{I}_{(200)}}\times 100$$

where *I*_*amorphous*_, and *I*_(200)_ represent the intensity of the amorphous halo and the intensity of the (200) peak, respectively. For the cellulose I, the at 2θ = 18° in the diffraction pattern was chosen as *I*_*amorphous*_. Crystallite size of the loaded MNPs were calculated using scherrer equation as given below in Eq. (2),

2 $$\tau =\frac{K\lambda }{\beta cos\theta }$$

where τ is the crystallite size, λ wavelength of X-rays, β full width of the peak at half minimum (FWHM) and θ is the scattering angle of the peak. XRD patterns were analyzed by Fityk software to calculate the FWHM values from the XRD patterns. Fourier transform infrared (FTIR) spectra of the samples were recorded in the range of 700–4000 cm^−1^ using PerkinElmer (spectrum100) ATR-FTIR spectrometer. X-ray photoelectron spectroscopy (XPS) Thermo Scientific K-Alpha KA1066 spectrometer (Germany) in the range of 200 to 1350 eV was investigated for the elemental analysis as well as for the determination of binding energy in the respective catalyst. The morphology of the samples was examined by field emission scanning electron microscope (FESEM) with a JEOL JSM-7600F, Japan. Energy dispersive X-ray spectrometry (EDS) of the samples was performed for the elemental analysis by using the Oxford-EDS system. Thermogravimetric analysis of 10 mg sample was performed by using TGA Q500 instrument. The samples were put in the aluminum pans and the autosampler stage of the instrument was kept on. Their weight lost against temperature under nitrogen atmosphere was recorded with a heating rate of 10 °C/min upto 700 °C. The residual concentration of nitrophenol and azo dyes was monitored and recorded by using Thermo scientific, Evolution 300 UV-visible spectrophotometer.

### Evaluation of catalytic activity

The catalytic activities of M^0^/CC-CH was evaluated in the reduction of nitrophenol (4-NP) and azo dye (Rh-B) by NaBH_4_. We used quartz UV cuvette cell as reaction vessel. We prepared three solutions of NaBH_4_, 4-NP and Rh-B with concentration of 0.1 M, 0.1 mM and 0.05 mM respectively, in DI water. As an example, we explain the typical procedure adopted for the conversion of 4-nitrophenol to 4-aminophenol by NaBH_4_ using the prepared catalyst. To UV cuvette, 3 mL of 4-NP and 0.5 mL of NaBH4 solution were added, and place in UV-visible spectrophotometer to record the UV-visible spectra. After this, the M^0^/CC-CH strip was added to this cuvette in such a way that UV-visible light can easily pass through it. The reduction reaction was started as soon as the catalytic strip was added and continuously recorded the absorption spectra. The recorded variation in the absorbance value of 4-NP at 400 nm was plotted and compared with the calibration curve. The catalytic strip was easily removed from the reaction vessel by just pulling the strip from cuvette and used it again for 4-NP conversion. The recyclability of the catalyst was performed after washing the catalytic strip with DI water and used again for 4-NP conversion reaction. The same procedure was implemented for the reduction reaction of Rh-B dye.

For sake of comparison, the catalytic activity of the bare CC and CC-CH was also tested. The catalytic efficiency of Fe/CC-CH for 4-NP and Rh-B was high as compared to other loaded MNPs, therefor we also investigated its activity towards other dyes like MB and MO. The concentration of these dyes was 0.05 mM. In all reaction, we used fresh catalytic strips except the recyclability experiments where same strip was used.

## Results and Discussion

**Figure 1:**
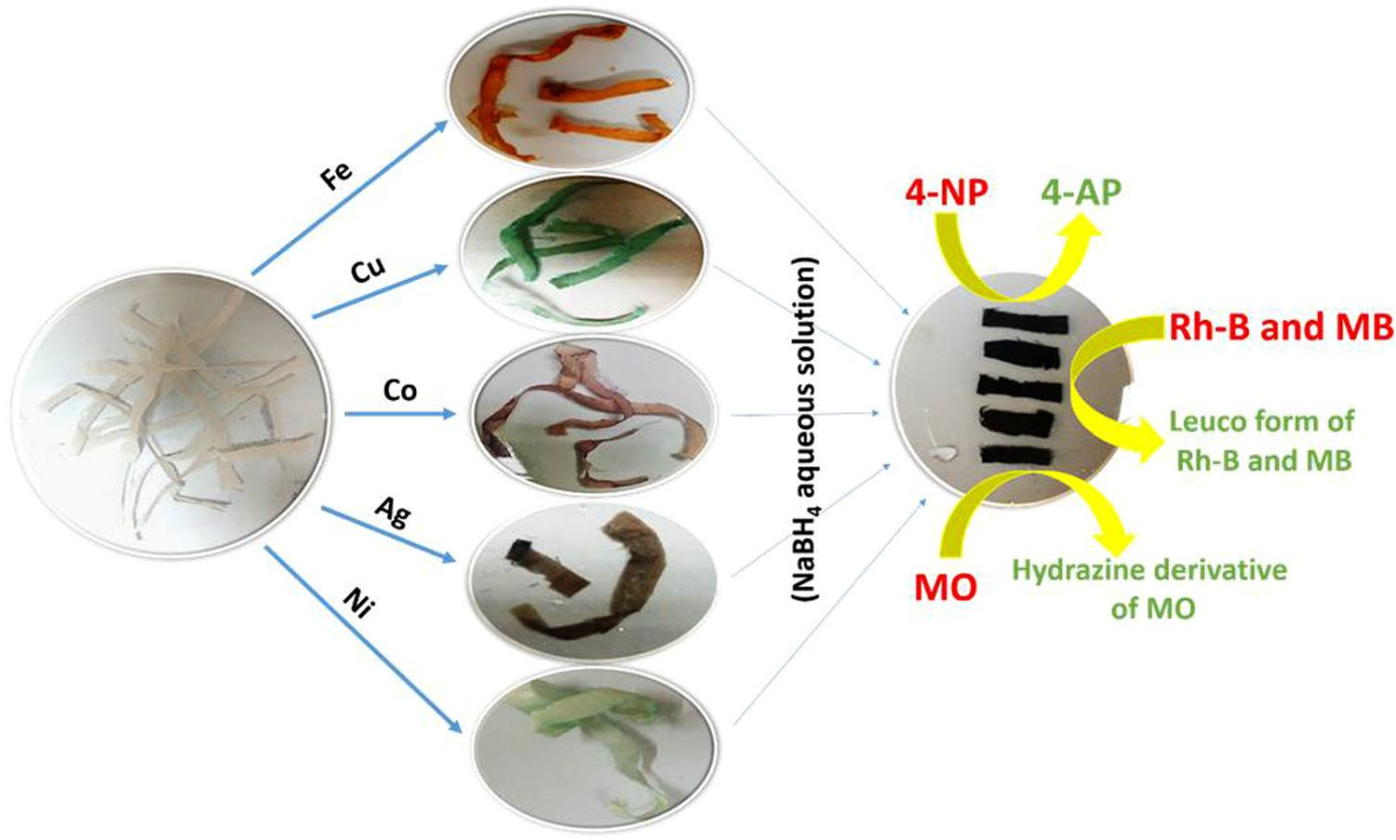
CC-CH pieces treated with Fe, Cu, Co, Ag and Ni salt aqueous solution, separately treated with NaBH_4_ aqueous solution after cutting in small equal strips and then these strips applied as catalyst for catalytic reduction of 4-NP, Rh-B, MB and MO solutions.

Figure 1 shows the steps involved in the preparation of M^0^/CC-CH catalytic strips. CC coating by CH resulted in mechanically strong and slightly brittle CC-CH as compare to the pure CC because the individual cellulose microfibers was connected by CH polymer. Then color of the CC-CH strips was change from white to yellowish, irish blueish, reddish, brownish and greenish after treating with aqueous solution of metal salt Fe, Cu, Co, Ag and Ni, respectively. The color change confirmed the uptake of metal ions by –NH_2_ and –OH groups present in CH layers of CC-CH strips. The formation of M^0^ was carried out by dipping the CC-CH loaded with metal ions in the aqueous solution of NaBH_4_ followed by color change to black. The color change took just few seconds, which indicates that the reaction of NaBH_4_ with metal ions was quite fast. The chemical reactions during this process are proposed as follow in Eq. (3–7):

3 $$2{{\rm{Co}}}^{2+}+4{{\rm{BH}}}_{4}^{-}+12{{\rm{H}}}_{2}{\rm{O}}\to 2{{\rm{Co}}}^{0}+4{\rm{B}}{({\rm{OH}})}_{3}+14{{\rm{H}}}_{2}$$

4 $$2{{\rm{Cu}}}^{2+}+4{{\rm{BH}}}_{4}^{-}+12{{\rm{H}}}_{2}{\rm{O}}\to {{\rm{2Cu}}}^{0}+4{\rm{B}}{({\rm{OH}})}_{3}+14{{\rm{H}}}_{2}$$

5 $$2{{\rm{A}}{\rm{g}}}^{+}+2{{\rm{B}}{\rm{H}}}_{4}^{-}+6{{\rm{H}}}_{2}{\rm{O}}\to 2{{\rm{A}}{\rm{g}}}^{0}+2{\rm{B}}{({\rm{O}}{\rm{H}})}_{3}+7{{\rm{H}}}_{2}$$

6 $$2{{\rm{Ni}}}^{2+}+4{{\rm{BH}}}_{4}^{-}+12{{\rm{H}}}_{2}{\rm{O}}\to 2{{\rm{Ni}}}^{0}+4{\rm{B}}{({\rm{OH}})}_{3}+14{{\rm{H}}}_{2}$$

7 $$2{{\rm{Fe}}}^{2+}+4{{\rm{BH}}}_{4}^{-}+12{{\rm{H}}}_{2}{\rm{O}}\to 2{{\rm{Fe}}}^{0}+4{\rm{B}}{({\rm{OH}})}_{3}+14{{\rm{H}}}_{2}$$

The successful synthesis of Co^0^, Cu^0^, Ag^0^, Ni^0^ and Fe^0^ nanoparticles depends on the water quality. In previous reports, water in NaBH_4_ aqueous solution was deoxygenated by using nitrogen or other inert gases to avoid metal oxide formation^33,34^. The M^0^ were also protected from their oxidation by the hydrogen gas release in the above reaction^7^. However, we used freshly prepared DI water for NaBH_4_ solution, where chances for dissolved oxygen was low. The prepared CC-CH strips loaded with different M^0^ were then tested in the reduction reactions of nitrophenol and dyes solutions.

### Crystal structure and morphology

#### XRD

**Figure 2:**
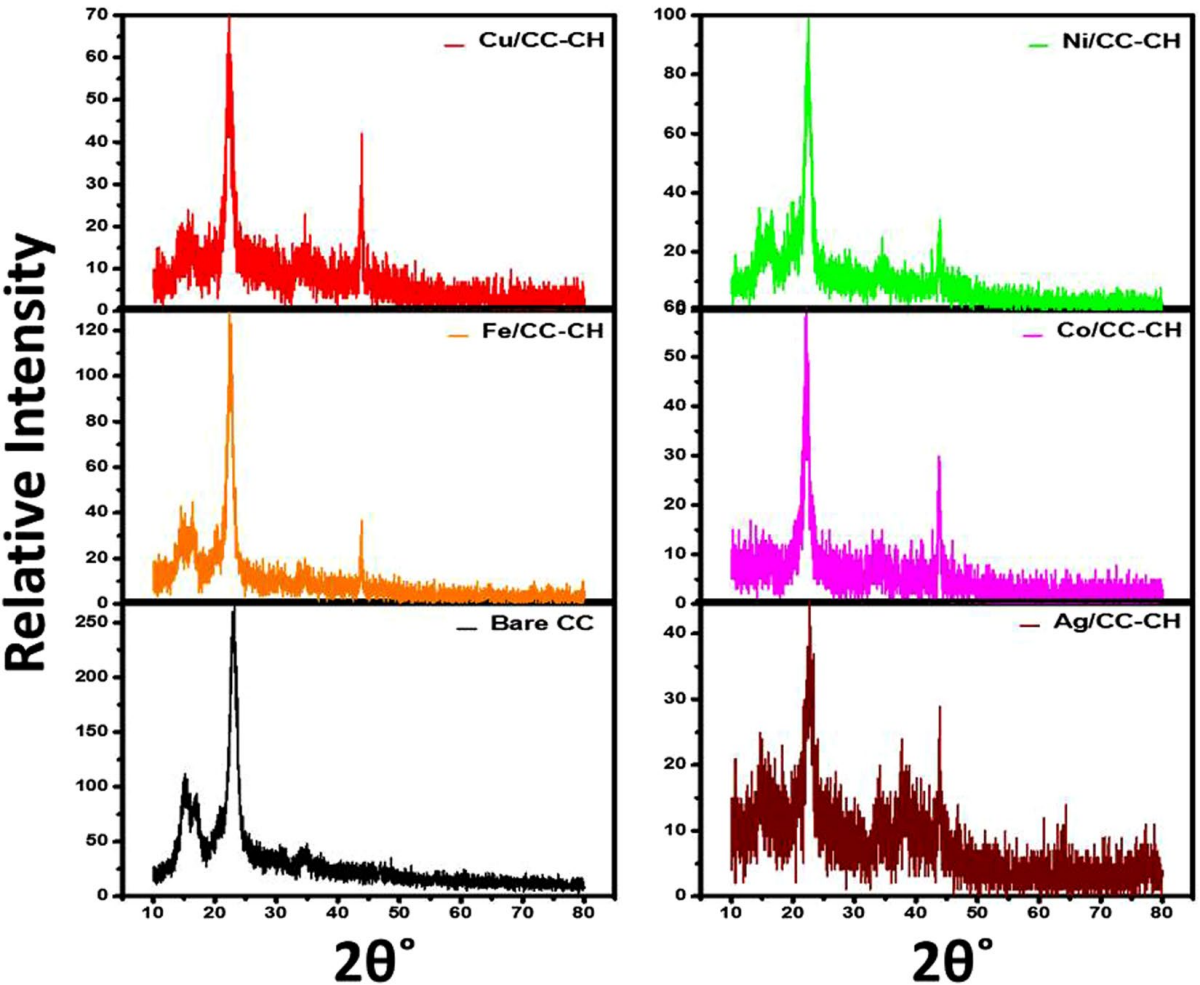
XRD patterns of bare CC and loaded CC-CH with Fe, Cu, Ag, Co and Ni nanoparticles.

Figure 2 shows the XRD patterns of the bare CC, and the loaded CC-CH with Fe, Cu, Ag, Co and Ni nanoparticles. All the patterns have diffraction peaks at 14.9°,16.7°, 22.9° and 34.7° can be observed which correspond to (110), (110), (200) and (004) planes of cellulose 1. The XRD pattern of the loaded CC-CH with Fe, Cu, Ag, Co and Ni were identical to the bare CC, which suggest that crystalline cellulose content was present in all samples and CH was only amorphously adsorbed on the CC. The crystallinity of all the samples were in the range of 70 to 77%, and the crystallite size was about 6.92 to 7.03 nm. The cellulose crystallinity and crystallite size of the MNPs loaded CC-CH were almost similar to the bare CC. These observations manifested that cellulose reserved its structure after processing with CH for the preparation of MNPs, which is of intense need for its mechanical properties. In addition to amorphous and crystalline peaks of cellulose fibers of CC, the sharpest peak of Fe nanoparticles was also observed at 2θ = 43.7°, corresponding to (400) plane of Fe nanoparticles according to JCPDS no 98–000–0064, which is in line with other reported literature^35^. Cu nanoparticles has a sharpest peak at 2θ = 43.72° corresponding to (111) reflection, according to the JCPDS card # 04–0836, which can be observed in the XRD pattern of Cu/CC-CH. Similarly, in the XRD pattern of Ag/CC-CH, we can observe the peaks at 2θ = 38 and 43.8°, corresponding to the (111) and (200) reflection planes and were in good agreement with previous reports^36,37^. Moreover, according to JCDPS, card no 15–0806, Co nanoparticles has a diffraction peak at 43.8° corresponding to (111) crystal plane, which was observed here in Co/CC-CH indicating the preparation of cubic crystalline cobalt. Actually two modification of Co coexist at room temperature, which is face-center cubic (FCC) and hexagonal close-packed (HCP), and are often difficult to separate^38^. Here, the prepared Co nanoparticles were determined to be FCC by JCPDS database, since in the reported pattern the FCC crystal structure was dominant. Also, no cobalt oxide or carbide phase was observed in the reported pattern, since their peaks are at 2θ = 42.4° (JCPDS card no. 09–0402) and 47.57° (JCPDS card no. 05–0727), respectively. In the Ni/CC-CH diffraction pattern, the peak at 2θ = 44° confirms the presence Ni nanoparticles of (111) plane (according to JCPDS file no. 04–0850), in addition to the cellulose peaks. Since, in all XRD patterns of MNPs loaded on CC-CH, no other peaks related to their oxide or hydroxide was observed, which suggest the successful synthesis of zero-valent MNPs in the layers of CH on CC.

#### FTIR

**Figure 3:**
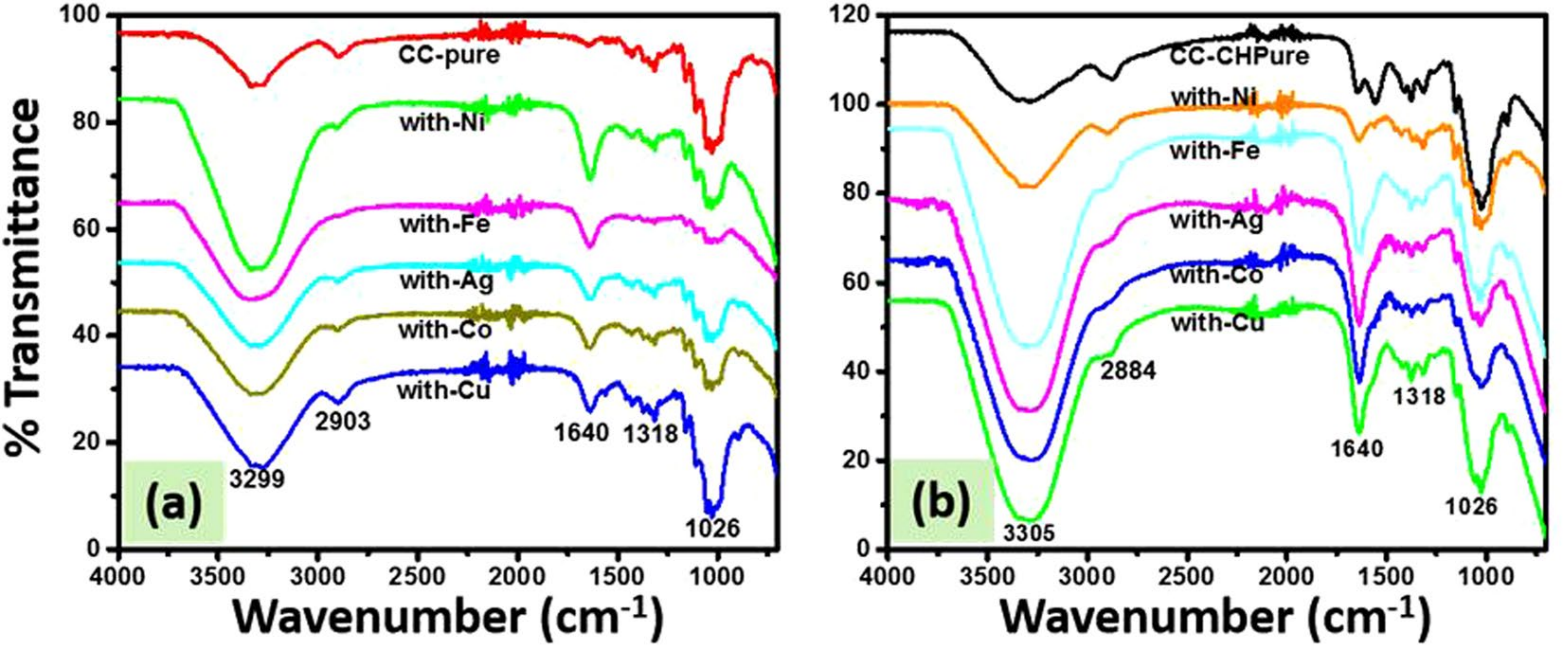
FTIR spectra of bare CC and loaded with Ni, Fe, Ag, Co and Cu nanoparticles (**a**), and bare CC-CH and loaded with Ni, Fe, Ag, Co and Cu nanoparticles (**b**).

Figure 3a,b shows the FTIR spectra of all the samples (bare CC and CC-CH and loaded with MNPs) recorded in the range between 700 cm^−1^ and 4000 cm^−1^. This figure suggests that all the other spectra were identical to those of pure CC spectrum. All the spectra exhibited a typical cellulose FTIR, which was widely described in previous literature^39,40^. Also, its peaks at are well assigned and discussed in previous reports^41,42^. FTIR analysis suggests that no interaction occur between oxygenated functional groups of CC, CH amino and oxygenated groups, and MNPs because in all spectra the peaks were located on the same position. The interaction of CH and MNPs with CC in low extent might be due to low content of CH and MNPs in the samples.

#### XPS

**Figure 4:**
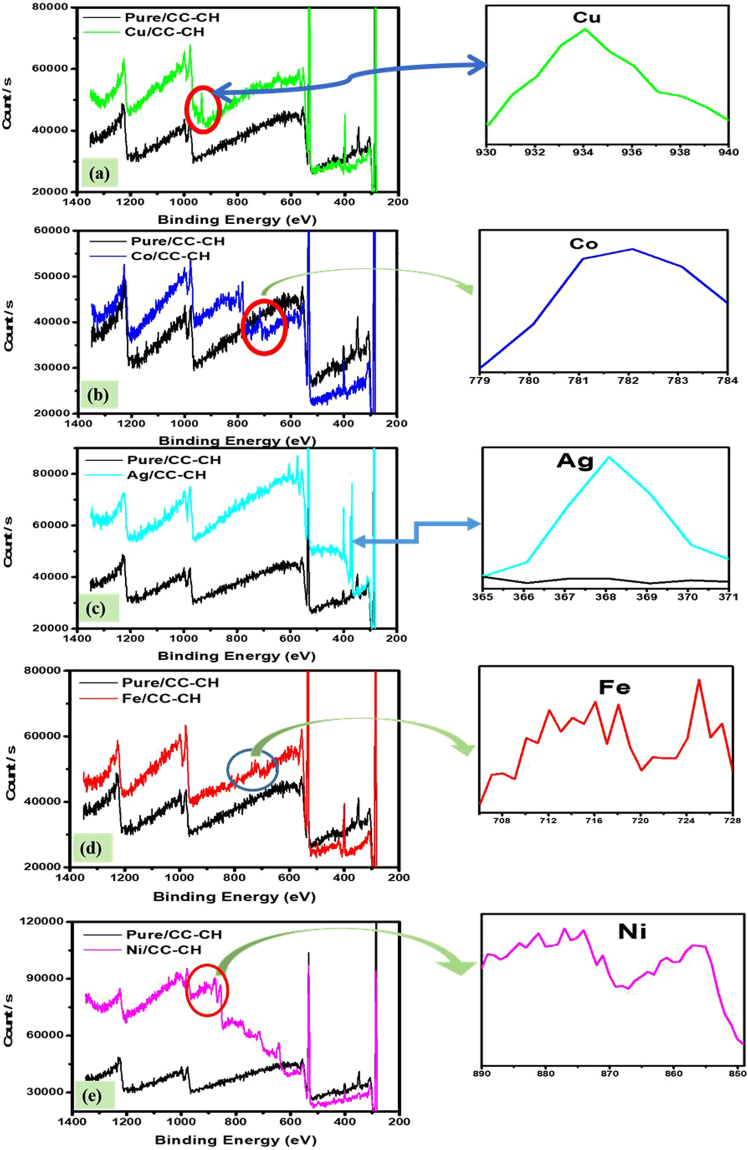
Comparison of XPS wide-scan survey of bare CC-CH and CC-CH coated with Cu (**a**), Co (**b**), Ag (**c**), Fe (**d**) and Ni (**e**) nanoparticles and their high resolution, respectively.

The XPS analysis of MNPs located on CC-CH surface in comparison with unloaded CC-CH is manifested in Fig. 4a–e. The valence state of the metals on the CC-CH surface is in the reduced form. For instance, Fig. 4a show the XPS Cu 2p_3/2_ survey, presenting a photoelectron peak, this peak is selected to the chemically reduced Cu^0^, based on the additional Auger line survey. However, for the survey of Co/CC-CH, our XPS analysis did not confirm that loaded Co nanoparticles was present in its zero-valent form. Rendering to this analysis, the recorded Co 2p_3/2_ peaks were positioned at 781 ± 0.3 eV and towards the high binding energy side. This value happens in between the earlier reported values correspond to Co^2+^ in Co(OH)_2_ (782 ± 0.1 eV for the 2p_3/2_ line)^43^ and to Co^2+^ in CoO (780 ± 0.2 eV for the 2p_3/2_ line)^44^. This line is very closer to the corresponding metallic cobalt, Co^0^ (778 ± 0.2 eV for the Co 2p_3/2_ line)^45^. Based on this, it could be proposed that Co^2+^ ions were simply precipitated on surface of CC-CH in the form of Co(OH)_2_ or fixed by the hydroxyl groups at that surface. Nevertheless, the XPS survey of Ag 3d_5/2_, has exposed only one valence state on the surface, which represent the reduced MNP, i.e., Ag^0^ (Fig. 4c). Thus, Ag^0^ nanoparticles are reserved on the CC-CH surface by chemical reduction and precipitation. For the survey of Fe 2p core levels (Fig. 4d), the photoelectron peaks at 711 eV, 719 eV and 725 eV epitomize the binding energies of Fe(2p_3/2_), shake-up satellite 2p_3/2_ and 2p_1/2_, respectively. These three main peaks of Fe suggest that the surface CC-CH strip mainly consist of iron nanoparticles^46,47^. Furthermore, a small shoulder at around 706.98 eV suggesting the 2p_3/2_ peaks of zero-valent iron (Fe^0^). The Ni 2p_3/2_ survey offers a small shoulder peak at 852 and another peak at 855 eV. That is, both Ni^0^ (852 eV) and Ni(II) (855 eV) are present on the CC-CH surface^47^. The presence of both the zero-valent and their oxidized form of nanoparticles might be due to exposure for long time to air atmosphere during XPS analysis.

#### FE-SEM and EDX

**Figure 5:**
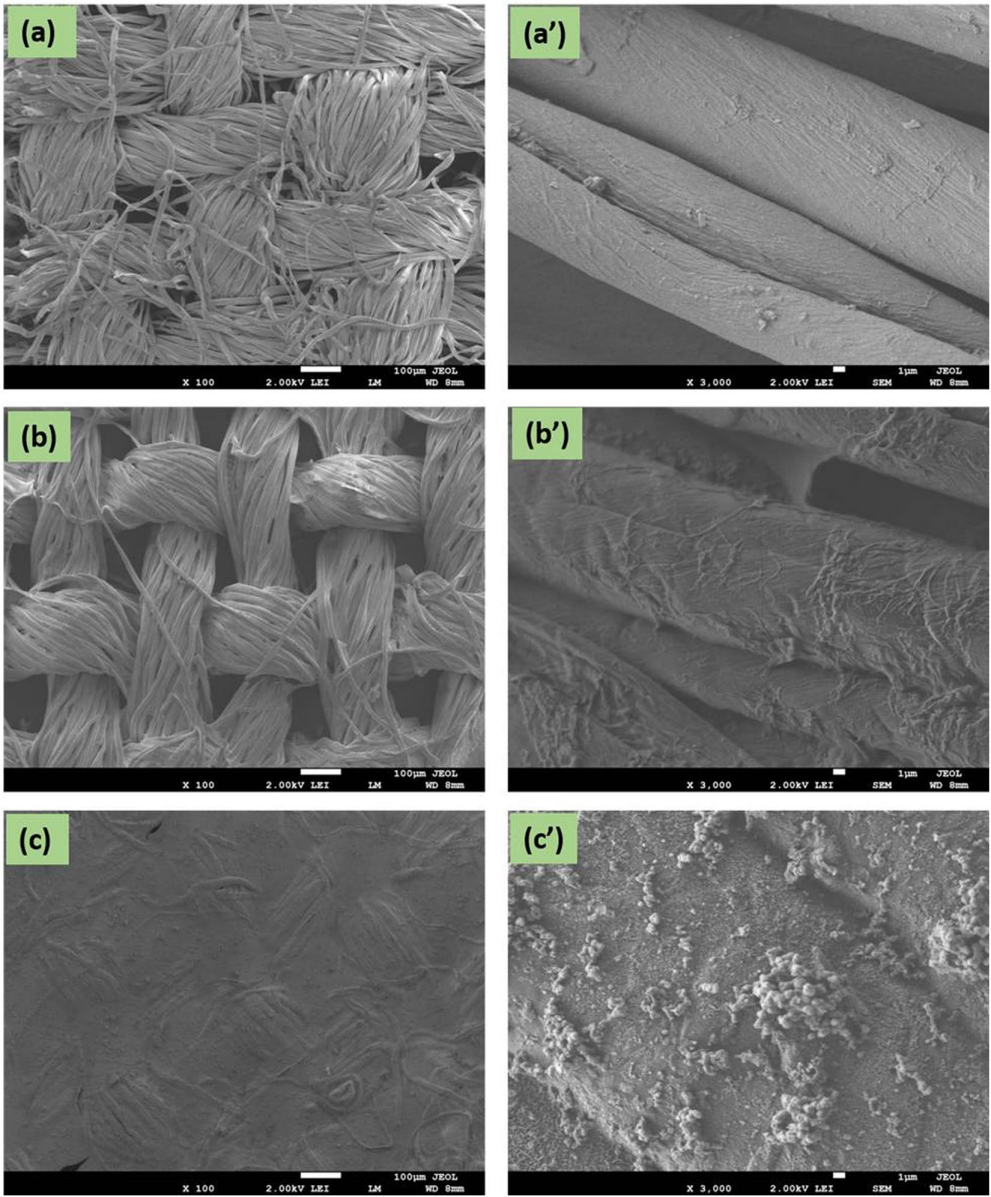
FE-SEM image of CC (**a**), CC-CH (**b**) and Fe/CC-CH (**c**) of low agnification and their respective high magnification (**a’–c’**).

**Figure 6:**
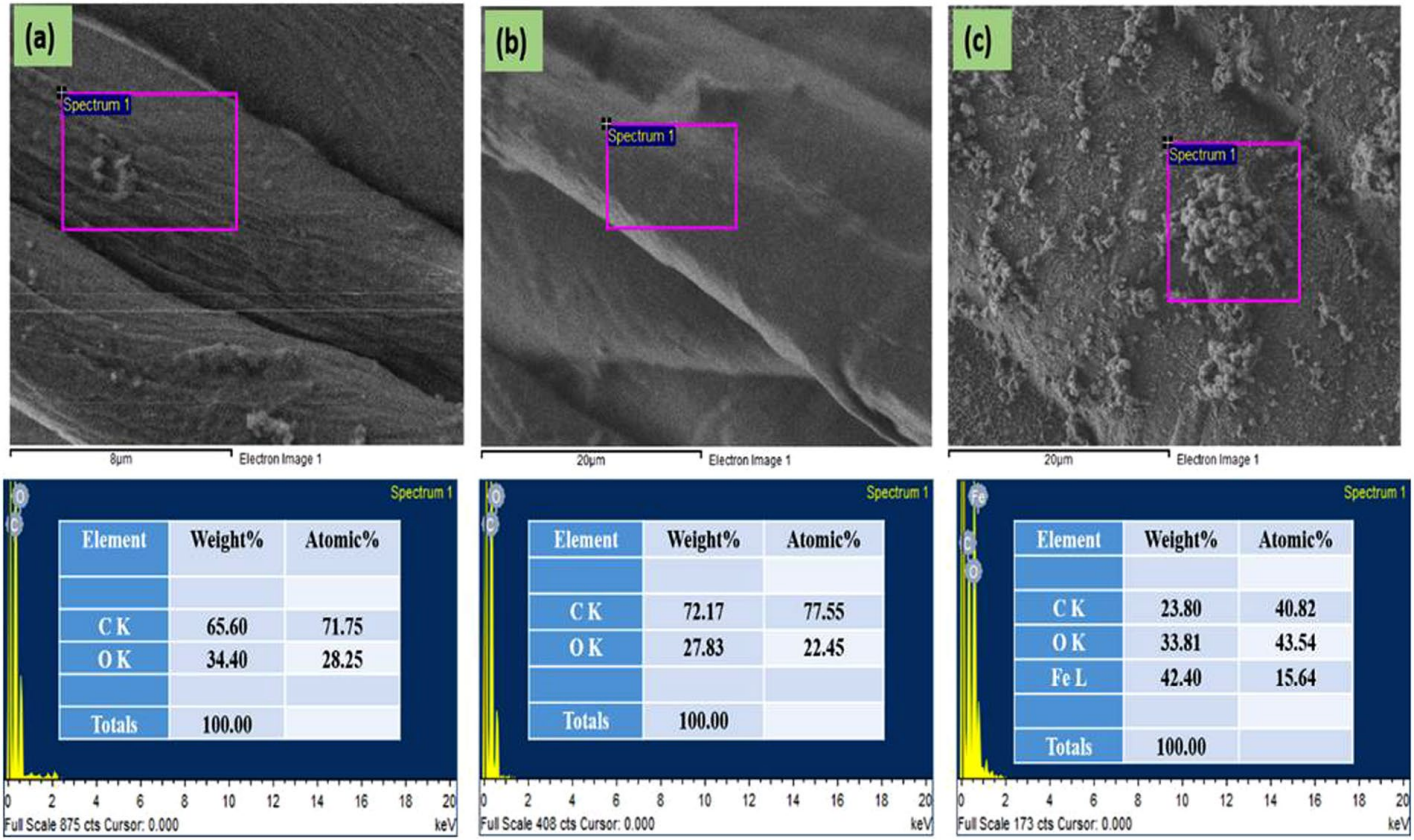
EDX spectra of CC (**a**), CC-CH (**b**) and Fe/CC-CH (**c**).

We used FE-SEM to analyze the morphologies of CC, CC-CH and Fe/CC-CH. Figure 5a–c exhibits the SEM images of all the samples. The left side images in (a–c) low magnification and the right side (a’–c’) exhibits high magnification. The FE-SEM image of CC was composed of microfibers as manifested in (Fig. 5a). The original morphology of CC upon treating with 5 wt% CH solution did not change (Fig. 5b). CC-CH was morphologically identical to the untreated CC and there were micropores in it. Initially, individual microfibers of CC are covered by a thin CH layer, as reveals by the images in (aa’) and (bb’). The CH thin layer are partly connecting the fibers, however, some of the fibers are separated from each other, which pointing to the fact that this layer might be wrapping these fibers. This result suggests that CH was adsorbed in the form of thin layer around the CC microfibers. In case of Fe/CC-CH, a significant morphological changes can be observed at micrometer scale. From Fig. 5c plenty of small spots can be observed on microfibers, which indicate that Fe nanoparticles have been successfully formed on the surface of CC-CH, as already confirmed by XRD analysis. Some aggregates were also observed in the SEM image at high resolution (Fig. 5c’). Beside some aggregation most of the CC fibers surface was covered with well separated and uniform Fe nanoparticles. The successful synthesis of Fe nanoparticles on CH layer over CC microfibers was also confirmed by EDS analysis (Fig. 6a–c). The elemental Fe can be clearly observed in the EDX spectrum of Fe/CC-CH (Fig. 6c). Other elements carbon and oxygen observed in EDS analysis were mostly due to the presence of various functional groups in CC and CH.

### TGA

**Figure 7:**
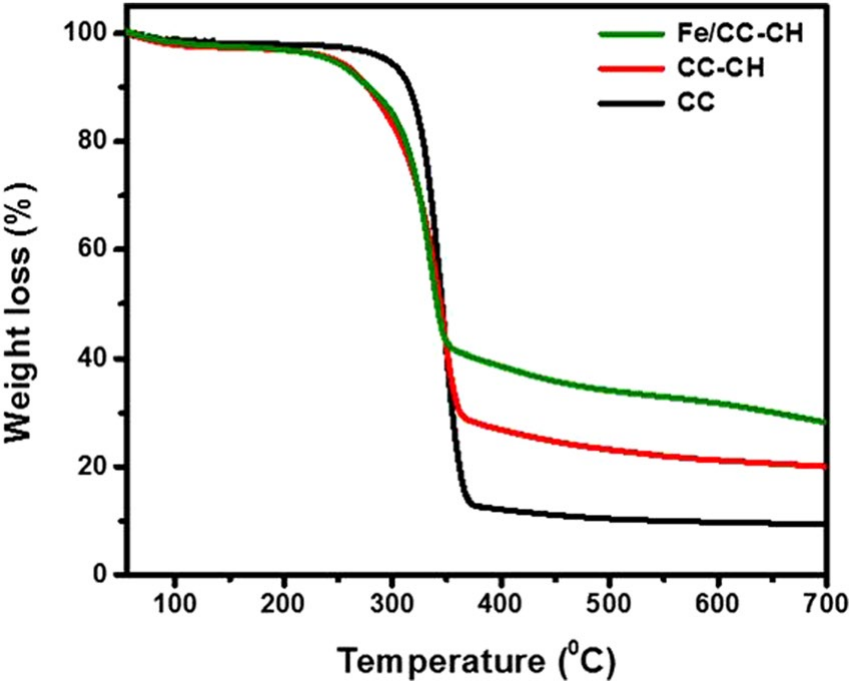
TGA thermogram of CC, CC-CH and Fe/CC-CH.

TGA thermograms of CC, CC-CH and Fe/CC-CH were elaborated in Fig. 7. All the sample lost around 10% of their original weight while increasing the temperature to 100 °C. The initial weight loss was due to the abolition of moisture from the samples. Moreover, an apparent and huge weight loss can be observed between 260 °C and 360 °C. This reduction of weight was due to oxidation, depolymerization and evolution of gases from cellulose and CH main organic portions. In contrast to other reports on addition of nanomaterial to polymer matrices, which increases the degradation temperature of polymer, a lowering in thermal stability of Fe/CC-CH was observed, which might be due to the catalytic nature of Fe nanoparticles. Usually nanoparticles increase the depolymerization and decreases the activation energy during thermal process^29,48,49^. Thermal decomposition temperature data obtained from TGA spectrum of CC, CC-CH and Fe/CC-CH are manifested in Table 1. In this table, (T_onset_) is the initiation of the thermal decomposition temperature at which cellulose microfibers start to decompose, (T_max_) is the maximum degradation rate temperature and (T_end_) is the decomposition end temperature. From Table 1, it is clear that T_onset_, T_max_ and T_end_ were slightly lower for Fe/CC-CH and CC-CH than CC. Another objective of TGA analysis was to determine the amount of Fe nanoparticles in Fe/CC-CH strip. The difference between Fe/CC-CH and CC-CH thermogram at 700 °C at 7.77%, which suggest that the strip of almost 10 mg of Fe/CC-CH contain 0.7 ± 0.5 mg of Fe nanoparticles.

**Table 1:** Important parameters calculated from TGA analysis of CC, CC-CH and Fe/CC-CH.

Samples	T_onset_ (^o^C)	T_max_ (^o^C)	T_end_ (^o^C)	Weight loss (%) at 700 ^o^C
CC	229	339	371	9.48
CC-CH	194	334	362	20.40
Fe/CC-CH	190	325	351	28.17

### Catalytic Property

#### 4-NP transformation to 4-AP

The catalytic activity of the different M^0^ template on CC-CH strips was analyzed by using it as a catalyst mainly in two (4-NP and Rh-B) degradation reactions. First, we studied the 4-NP transformation to 4-aminophenol (4-AP) in the presence of prepared catalytic materials. The catalytic conversion of 4-NP to 4-AP has been extensively studied from the last few years, because it may be an efficient and greener method for the preparation of 4-AP^50–52^. 4-AP is an efficient intermediate and raw material for the preparation of several medicinal and cosmetic products^53,54^. Besides, it is reported that 4-AP is less toxic than nitroaromatic compounds and can be easily mineralized and removed from the environment^55,56^. Therefore, catalytic conversion of 4-NP to 4-AP is an efficient process and when analysis of catalytic activity of catalyst is required this reaction is often chosen as a model reaction.

**Figure 8:**
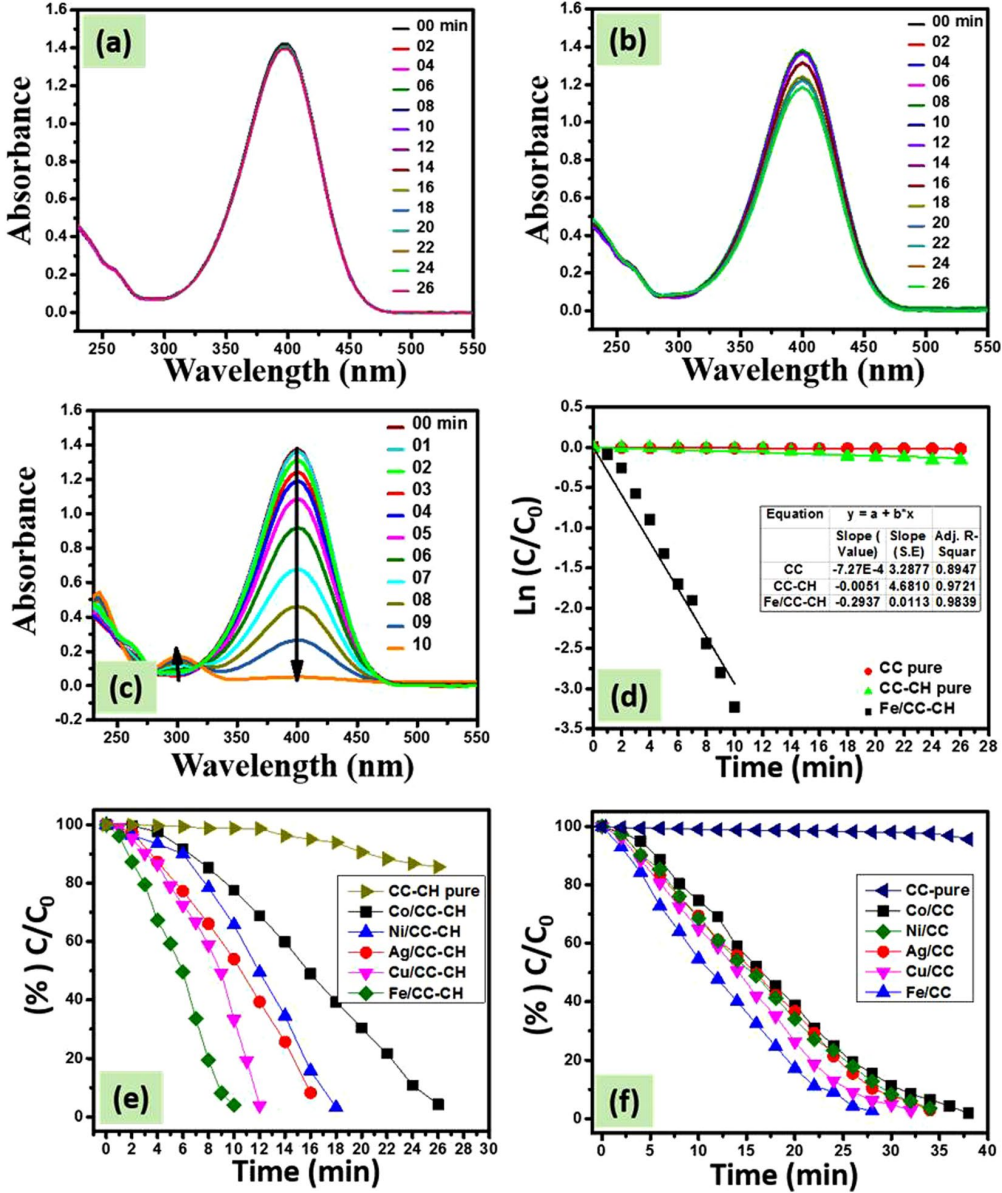
UV-vis spectra of 4-NP reduction as a function of time by NaBH_4_ in the presence of bare CC (**a**), bare CC-CH (**b**) and Fe/CC-CH (**c**), Ln(C/C_0_) vs time for the reaction occur in the presence of bare CC, CC-CH and Fe/CC-CH (**d**), percent C/C_0_ of 4-NP as a function of time by NaBH_4_ in the presence of bare CC-CH and loaded with Fe, Cu, Ag, Ni and Co nanoparticles (**e**) and bare CC loaded with Fe, Cu, Ag, Ni and Co nanoparticles (**f**). Experimental conditions: 3 mL of 0.1 mM 4-NP solution, 0.5 mL of 0.1 M NaBH_4_ aqueous solution and 0.4 × 2 cm^2^ of the respective catalytic strip.

A CC strip of dimension 0.4 × 2 cm^2^ was introduced in UV cuvette containing 3 mL of 4-NP aqueous solution and 0.5 mL of NaBH_4_. No change in color of the solution was observed. Moreover, by analyzing UV-visible spectral data before and after 26 min insertion of CC, a slight decrease in the intensity was observed at 400 nm (main broad peak) (Fig. 8a). Similarly, the incorporation of CC-CH, slightly changes the color of 4-NP and its UV-visible spectra (Fig. 8b). Such a slight decrease in intensity might be due to the physical adsorption of 4-NP by CC or CH, which also indicates that pure CC and CC-CH have no catalytic property. Contrary to bare CC and CC-CH strips, when M^0^ loaded strips were introduced to the reaction matrix of same conditions, the disappearance of color of the solution was observed as shown in Fig. SI 1. The intensity of the peak at 400 nm was gradually disappeared and completely vanished after 10, 12, 16, 18 and 26 min, respectively in the presence of CC-CH loaded with zero-valent Fe, Cu, Ag, Ni and Co nanoparticles (Fig. 8c and Fig. SI 2a–d). In this catalytic reduction reaction of 4-NP, the resulted increase in a new absorption band at 300 nm is recognized as the formation of 4-AP. Probably, the mechanism of this reaction remains the topic of discussion because it may involve complicated intermediates located on the surface of the NPs^57,58^. However, the isosbestic point approximately at wavelength of 320 nm exhibits the complete conversion 4-NP to 4-AP without side reaction^58,59^. The CC loaded with Fe, Cu, Ag, Ni and Co nanoparticles took 28, 32, 34, 34 and 38 min for complete reduction of 4-NP aqueous solution (Fig. 8f). In comparison of percent remaining concentration (%C_t_/C_0_) of nitrophenol versus time in the presence of CC and CC-CH loaded with different M^0^, it can be observed that Fe/CC-CH exhibited good catalytic efficiency (Fig. 8d and f), which suggests that Fe-NPs are more active towards nitrophenols reduction. These results strongly support the superior and excellent catalytic activity of the Fe nanoparticles template in thin CH layers over CC. A small induction period can be revealed over all catalytic strips. Generally, it is considered that the induction period is due to the diffusion of 4-nitrophenolate ions onto the surface of catalyst to be adsorbed prior to the start of the reaction^60^.

The 4-NP reductive conversion to 4-AP in the presence of strong reducing agent NaBH_4_, is six electron conversion process^32,50^. The conversion of 4-NP to 4-AP is thermodynamically favorable in the presence of NaBH_4_, because their standard electrode potential is greater than zero, *i.e* 0.67 V^32,61^. However, this conversion reaction is kinetically unfavorable in the absence of suitable and efficient catalyst as evidenced experimentally by the use of CC and CC-CH strips. The kinetic barrier can be by-passed through the placement of suitable catalyst like in the present case demonstrated as supported MNPs. It was previously demonstrated that introduction of NaBH_4_ in nitrophenols produce nitrophenolate ions, the borohydrides ions (BH_4_^−^) and nitrophenolate ions get adsorbed on the surface of the catalyst for transfer of electrons. Most of the time, these reduction reactions proceed with *pseudo-first-order* kinetics, provided that NaBH_4_ is used in excess amount^32,60^. The natural log of absorbance ratios *(ln(C/C*_0_)*)* of the peak at 400 nm was plotted against time for 4-NP aqueous solution (Fig. 8d), where CC, CC-CH and Fe/CC-CH were used as a catalyst. All the data in the figure of *Ln(C/C*_0_ *)* vs time followed linear equation, which suggests that the reaction progressed with *pseudo-first-order* kinetics. The rates of reaction were 0.0007, 0.0051 and 0.2937 min^−1^ for CC, CC-CH and Fe/CC-CH.

**Figure 9:**
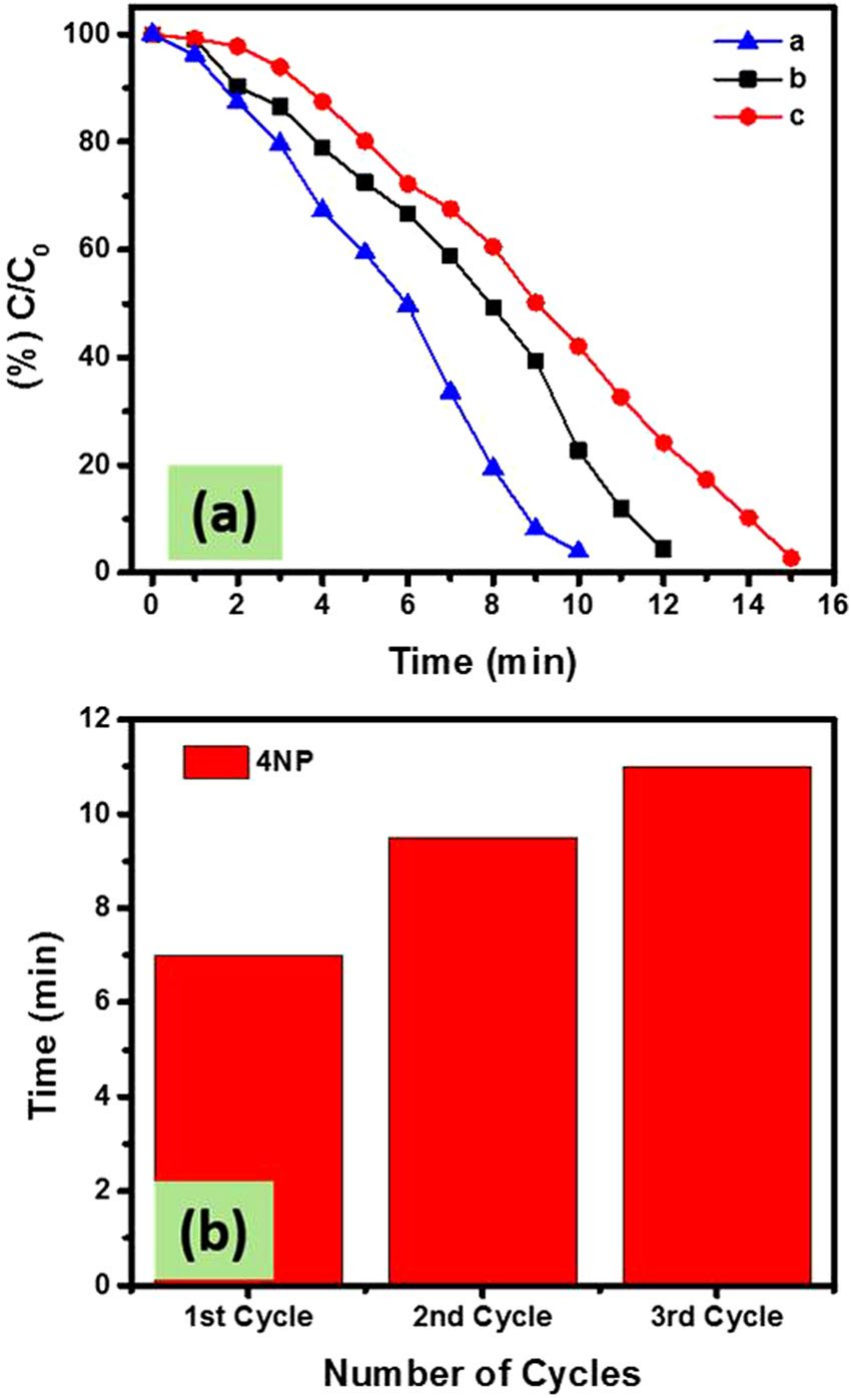
Percent reduction of 4-NP by NaBH_4_ in the presence of same catalytic strip Fe/CC-CH re-used three times (**a**), time taken by recycling catalyst Fe/CC-CH for 70% reduction of 4-NP (**b**).

Although metal nanoparticles were reported as an effective catalyst for the conversion of 4-NP to 4-AP, but they are difficult to separate from reaction matrix and to re-use for next cycle. Therefore, for reusability purpose the preparation of easy separable catalyst is rather essential. The M^0^/CC-CH catalytic strip can be easily pulled out from the reaction matrix. After one time use of the catalytic strip of Fe/CC-CH in 4-NP to 4-AP reaction, the same strip was washed with DI water and used in new reaction of 4-NP. The plot of (%) C_t_/C_0_ as function of time, while using the same strip of Fe/CC-CH for 4-NP reduction is manifested in Fig. 9a. It can be observed that more than 95% of 4-NP was transformed into 4-AP during three time use of the same catalytic strip. The time taken by re-using the same catalytic strip of Fe/CC-CH for 70% reduction of 4-NP can be seen from Fig. 9b. The increase in reduction time was observed using the same catalytic strip, which indicated the diminution in catalytic performance. Such a decrease in the catalytic performance might be due to the oxidation of Fe nanoparticles during handling and their slight release to the solution matrix.

#### Rhodamine-B reduction

**Figure 10:**
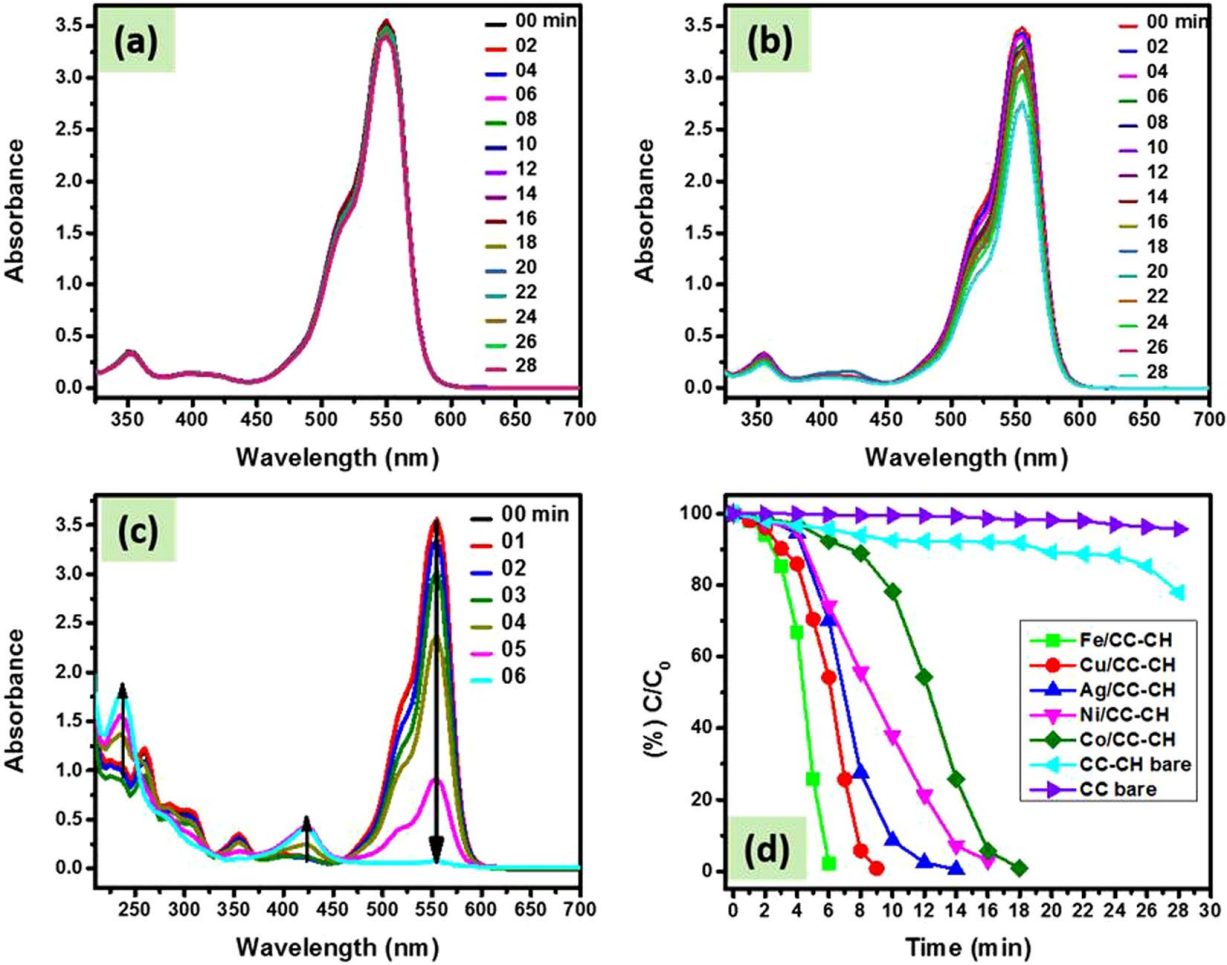
UV-vis spectra of Rh-B reduction as a function of time by NaBH_4_ in the presence of bare CC (**a**), bare CC-CH (**b**), Fe/CC-CH (**c**) and percent C/C_0_ of Rh-B as a function of time by NaBH_4_ in the presence of bare CC, bare CC-CH and CC-CH loaded with Fe, Cu, Ag, Ni and Co nanoparticles (**d**). Experimental conditions: 3 mL of 0.05 mM Rh-B solution, 0.5 mL of 0.1 M NaBH_4_ aqueous solution and 0.4 × 2 cm^2^ of the respective catalytic strip.

Same procedure was applied for Rh-B reduction as applied for 4-NP. Rh-B is one of the important xanthene dye and famous for its stability, which is widely utilized as a colorant in textiles and food stuffs^62,63^. It is harmful to human beings and causes irritation of the eyes, skin and respiratory tract. The reproductive and developmental toxicity, carcinogenicity, chronic toxicity and neurotoxicity have been experimentally proven toward humans and animals^64,65^. Keeping the harmful effects and hazardous nature of Rh-B in view, many efforts were made to degrade it from aqueous environment^66–68^. The UV-vis spectra of reduction of Rh-B as function of time are manifested in Fig. 10a–c and Fig. SI 3a–d. As was expected, a slight decrease was observed in the intensity at 552 nm for the bare CC and CC-CH in 28 min (Fig. 10a,b). Besides, the CC-CH loaded with M^0^ show catalytic efficiency toward Rh-B. The time taken for complete reduction of Rh-B was 18, 16 and 14 min in presence of CC-CH loaded with Co, Ni and Ag nanoparticles, while it took 9 and 6 min for Cu and Fe nanoparticles respectively. Inducing period existed in the presence of all catalyst for reduction reaction of Rh-B by NaBH_4_. However, the inducing periods were not identical, which show the difference in catalytic approach by different metal nanoparticles. The remaining percent of Rh-B was plotted against time in presence of various MNPs templated on CC-CH (Fig. 10d). It can be observed that Fe/CC-CH strip has high catalytic efficiency as compared to other loaded nanoparticles, which show the superior catalytic activity of Fe^0^ nanoparticles towards Rh-B.

#### MO and MB reduction

**Figure 11:**
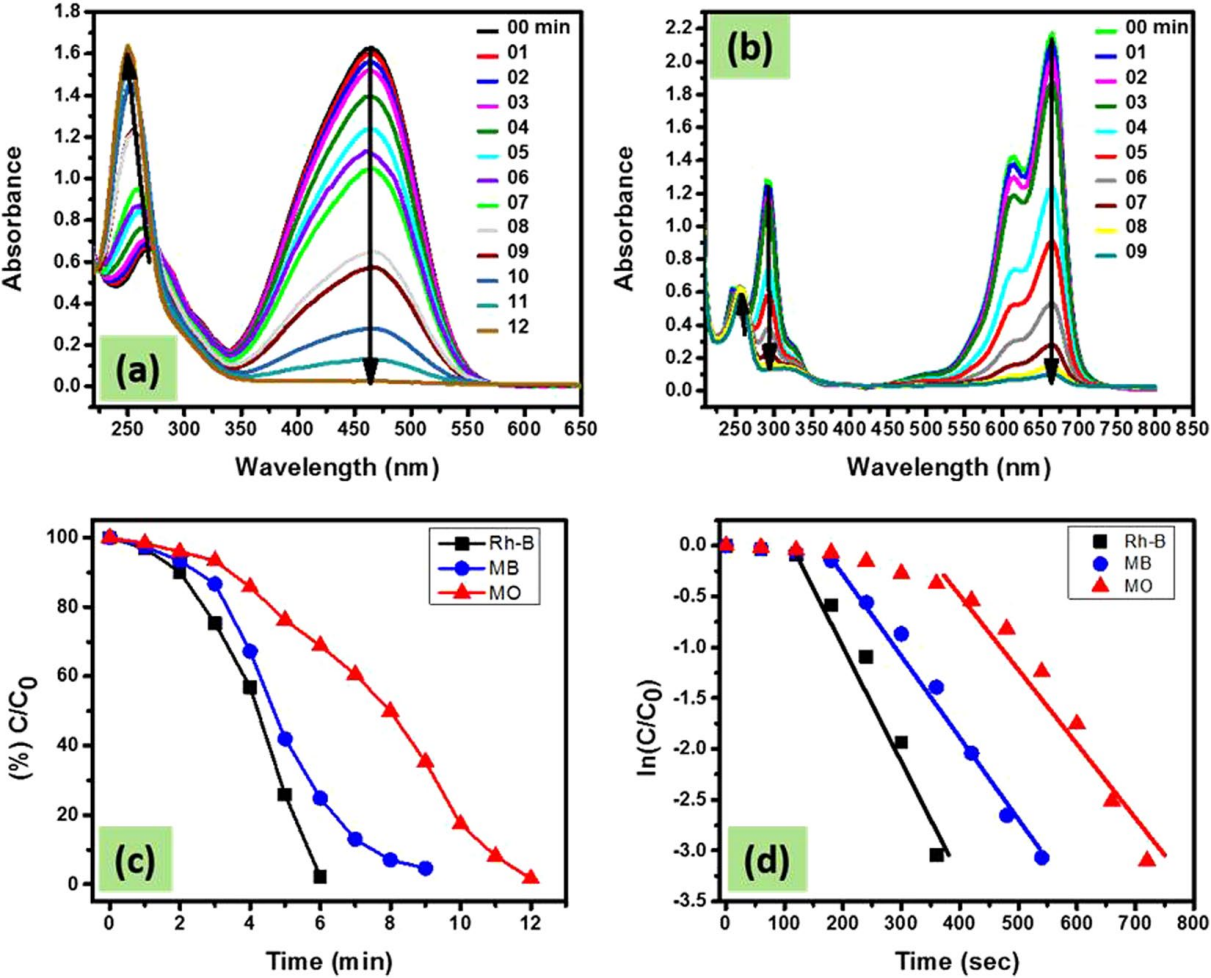
UV-vis spectra of MO (**a**) and MB (**b**) reduction as a function of time by NaBH_4_ in the presence of Fe/CC-CH catalytic strip, Percent C/C_0_ vs time for Rh-B, MO and MB (**c**), and and their Ln(C/C_0_) values as a function of time (**d**) Experimental conditions: 3 mL of 0.05 mM Rh-B or MO or MB solution with 0.5 mL of 0.1 M NaBH_4_ aqueous solution and 0.4 × 2 cm^2^ of the respective catalytic strip.

**Figure 12:**
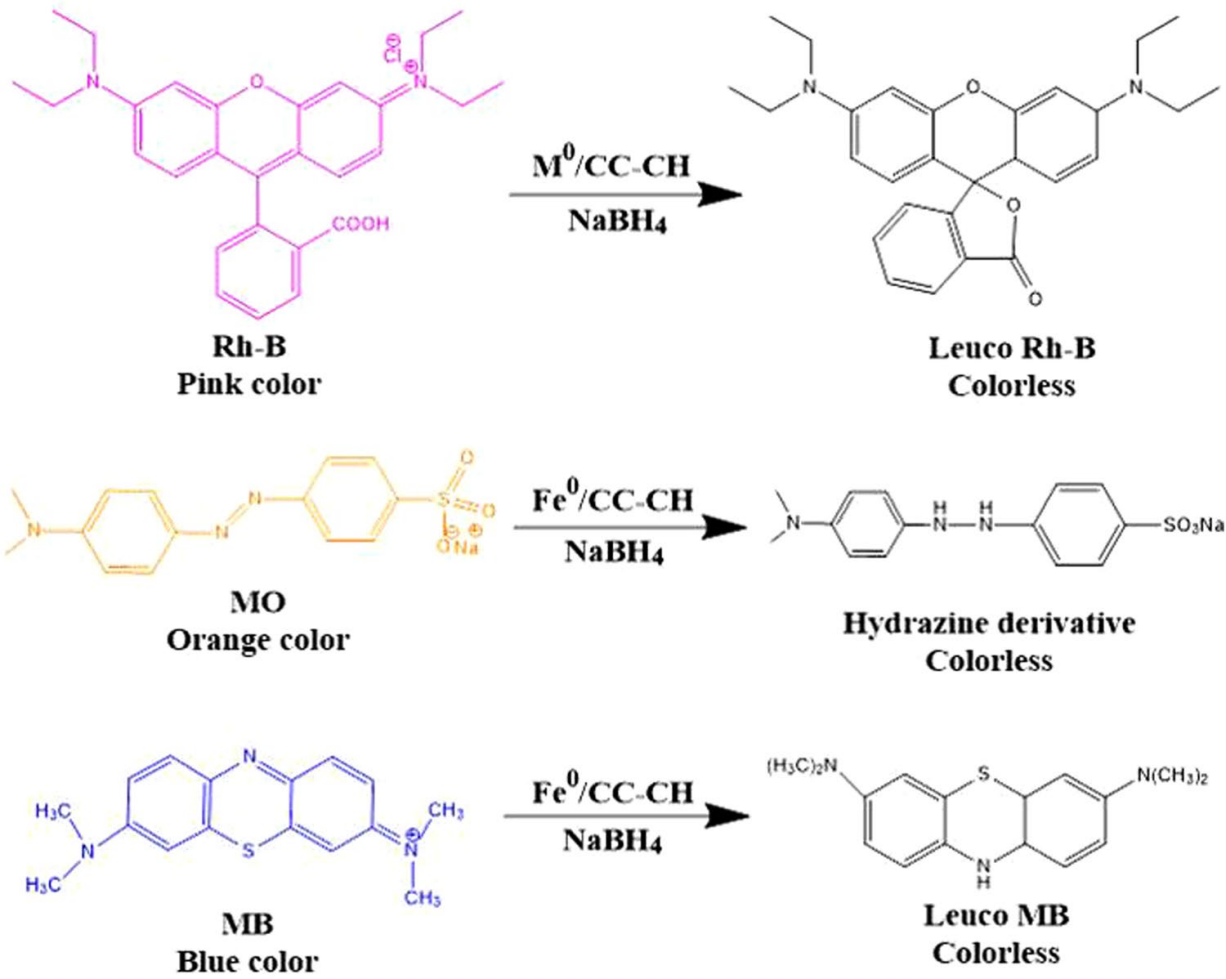
Reduction reactions of dyes to their leuco forms (hydrazine derivative of MO).

Beside Rh-B dye, we also investigated the catalytic affinity of Fe/CC-CH on other organic dyes like MO and MB in the above NaBH_4_ system. In the UV-vis absorption spectra of these dyes, the highest absorption peaks were observed at 464 for MO and 664 for MB. After adding NaBH_4_ solution in the cuvette containing dyes solution, the site of the absorption peak and intensity did not change, implying that between dyes and NaBH_4_ no reductive reaction took place. However, the peak intensities gradually decreased with time by introducing Fe/CC-CH strip to the above dye-NaBH_4_ system (Fig. 11a,b). Similar to the reduction of Rh-B, these dyes reduction was at slower rate in the start of the reaction called as incubation/inducing period. Obviously, the different change trend under the same reaction conditions should originates from difference in molecular structure of the dyes. In general, the electron transfer from the BH_4_^−^ ions onto the dyes molecules are responsible for the reduction of dyes, which take place with assistance of catalyst (here Fe/CC-CH)^69,70^. The overall process can be explained with electron relay system. The catalytic reduction started by Fe nanoparticles by transmitting electrons from the donor NaBH_4_ to the dye molecule. Actually, when NaBH_4_ was added to the reaction matrix, the Fe nanoparticles may be trapped by the hydride from BH_4_^−^ ions and adsorbed on the surface of Fe/CC-CH strip and transfer its electron to the Fe nanoparticles. After the electron transfer to the Fe nanoparticles from BH_4_^−^ ions the hydrogen atom formed, which subsequently attack a nearby dye molecule. Then spontaneously hydrogenation of the dye occurs by the transfer of these electrons to dye molecule. In other words, a negatively charges Fe nanoparticles (having excess of electrons), finally released electrons to an electron acceptor (here dye molecule), producing their reduced form (leuco Rh-B, leuco MB and hydrazine derivate for MO) as shown by the appearance of the new peaks around 250 nm in their time dependent UV-absorption spectra (Figs 10c,11a,b and 12). The cationic dyes (Rh-B and MB) reduced first followed by anionic dye MO. The nature of a dye (*via*., charge, presence of S/N donor atoms, hydrophobicity) may affect its rate of reduction^71,72^. In general, cationic dyes like Rh-B and MB have a high catalytic reaction rate then anionic dyes like MO, because of electrostatic interactions. It is clear that upon introduction of Fe/CC-CH catalytic strip in various dyes solution, the reduction reaction completed about 6, 9 and 12 min for RB, MB and MO, respectively. These suggests that Fe/CC-CH is responsible for the possible cleavage of -N = N- double bond in azo dye structure and thus decolorizing the solutions^73^. Also, the appearance of new peaks in the UV-vis absorption spectra indicated the formation of new products. From Fig. 11c, it can be clearly observed that Fe/CC-CH has high catalytic efficiency of reduction towards Rh-B. More detailed information was deduced from the kinetics of reduction reactions. In these experiments, the concentration of NaBH_4_ was much higher than dyes and could be consider as constant during the reaction kinetics. The order of the reaction was determined by *pseudo first order* kinetic equation, *Ln(C/C*_*0*_ *)* = *−kt*. The values of *Ln(C/C*_*0*_) versus time was plotted as shown in Fig. 11d. The values in the presence of Fe/CC-CH, didn’t follow fully linearity of the equation might be due to the inducing period in the start of the reaction and also the bubble formation in the cuvette during the reaction. The rate of reactions calculated after deducing the inducing periods were 0.00283, 0.00467 and 0.00634 sec^−1^ for MO, MB and Rh-B, respectively. The high rate constant value deduced the high catalytic nature of Fe/CC-CH strip toward Rh-B, which is much higher than the reported literatures^71,74^. Comparison of our work with literature for the reduction of 4-NP and azo dyes is manifested in Table 2.

**Table 2:** Comparison of catalysts for the reduction of 4-NP and azo dyes with literature.

Catalysts	Reduction reaction of	K_app_ (min^−1^)	References
Fe/CC-CH	4-NP	0.2937	This work
Cu/ZnPc-CH	4-NP	0.0513	25
Au/CA	4-NP	0.14–0.20	52
Ag/CB-CH	4-NP	0.1040	26
Ag/CH-FP	4-NP	0.2340	60
Au (10)/TiO_2_	4-NP	0.17	75
FeNi_2_ alloy nanostructure	4-NP	0.06	76
Fe/CC-CH	Rh-B	0.3804	This work
Bi_2_WO_6_	Rh-B	0.0517	77
Fe/CC-CH	MO	0.1698	This work
Ni/CH-FP	MO	0.116	32
Fe/CC-CH	MB	0.2802	This work
Ag/RGO	MB	0.014	78

## Conclusion

In summary, we present a simple method for the preparation of MNPs in the CH coating layer over cotton cloth for catalytic purpose. FTIR, XRD, XPS and TGA analysis confirmed that other properties of the cellulose fibers and CH did not change during the process. FESEM and EDX analysis exposed that the Fe nanoparticles were synthesized on CC-CH strips. Bare CC and CC-CH loaded with different MNPs were investigated for catalytic reduction of 4-NP and Rh-B by NaBH_4_. We showed that Fe/CC-CH strips have good catalytic activity for 4-NP and Rh-B as well as for MO and MB dyes reduction. The reduction reaction for 4-NP, Rh-B, MO and MB by NaBH_4_ in presence of Fe/CC-CH strip were accomplished within 10, 6, 12 and 9 min, respectively. The Fe/CC-CH was re-used for three time in the reduction reaction of 4-NP, where more than 90% reduction was achieved in 15 min. The separation of the catalytic strip was nicely done by simply pulling the strip from the reaction matrix.
